# Defining Disease, Diagnosis, and Translational Medicine within a Homeostatic Perturbation Paradigm: The National Institutes of Health Undiagnosed Diseases Program Experience

**DOI:** 10.3389/fmed.2017.00062

**Published:** 2017-05-26

**Authors:** Timothy Gall, Elise Valkanas, Christofer Bello, Thomas Markello, Christopher Adams, William P. Bone, Alexander J. Brandt, Jennifer M. Brazill, Lynn Carmichael, Mariska Davids, Joie Davis, Zoraida Diaz-Perez, David Draper, Jeremy Elson, Elise D. Flynn, Rena Godfrey, Catherine Groden, Cheng-Kang Hsieh, Roxanne Fischer, Gretchen A. Golas, Jessica Guzman, Yan Huang, Megan S. Kane, Elizabeth Lee, Chong Li, Amanda E. Links, Valerie Maduro, May Christine V. Malicdan, Fayeza S. Malik, Michele Nehrebecky, Joun Park, Paul Pemberton, Katherine Schaffer, Dimitre Simeonov, Murat Sincan, Damian Smedley, Zaheer Valivullah, Colleen Wahl, Nicole Washington, Lynne A. Wolfe, Karen Xu, Yi Zhu, William A. Gahl, Cynthia J. Tifft, Camillo Toro, David R. Adams, Miao He, Peter N. Robinson, Melissa A. Haendel, R. Grace Zhai, Cornelius F. Boerkoel

**Affiliations:** ^1^NIH Undiagnosed Diseases Program, Common Fund, Office of the Director, National Institutes of Health, Bethesda, MD, United States; ^2^National Human Genome Research Institute, National Institutes of Health, Bethesda, MD, United States; ^3^Department of Molecular and Cellular Pharmacology, University of Miami School of Medicine, Miami, FL, United States; ^4^Appistry, Inc., St. Louis, MO, United States; ^5^MicroSoft Research, Redmond, WA, United States; ^6^William Harvey Research Institute, Barts and The London School of Medicine and Dentistry, Queen Mary University of London, London, United Kingdom; ^7^Environmental Genomics and Systems Biology, Lawrence Berkeley National Laboratory, Berkeley, CA, United States; ^8^Palmieri Metabolic Disease Laboratory, Children’s Hospital of Philadelphia, Philadelphia, PA, United States; ^9^Department of Pathology and Laboratory of Medicine, University of Pennsylvania, Philadelphia, PA, United States; ^10^The Jackson Laboratory for Genomic Medicine, Farmington, CT, United States; ^11^Department of Medical Informatics and Clinical Epidemiology, Oregon Health & Science University, Portland, OR, United States

**Keywords:** rare disease, human phenotype ontology, distributed cognition, diploid alignment, glycome

## Abstract

Traditionally, the use of genomic information for personalized medical decisions relies on prior discovery and validation of genotype–phenotype associations. This approach constrains care for patients presenting with undescribed problems. The National Institutes of Health (NIH) Undiagnosed Diseases Program (UDP) hypothesized that defining disease as maladaptation to an ecological niche allows delineation of a logical framework to diagnose and evaluate such patients. Herein, we present the philosophical bases, methodologies, and processes implemented by the NIH UDP. The NIH UDP incorporated use of the Human Phenotype Ontology, developed a genomic alignment strategy cognizant of parental genotypes, pursued agnostic biochemical analyses, implemented functional validation, and established virtual villages of global experts. This systematic approach provided a foundation for the diagnostic or non-diagnostic answers provided to patients and serves as a paradigm for scalable translational research.

## Introduction

As established in 2008, the purpose of the National Institutes of Health (NIH) Undiagnosed Diseases Program (UDP) is to provide answers to patients with conditions that have eluded diagnosis and to advance medical knowledge about rare and common diseases (1). At its fundamental core, the NIH UDP is, therefore, an implementation of both personalized and genomic medicine.

Personalized medicine, which is the customization of healthcare to the individual patient, conceptually flows from the dawn of medicine. Medical practice has had a long tradition of being inherently “personal” to each patient. Current usage of “personalized medicine” denotes, however, the use of technology to enable a personalization not previously feasible and is generally applied in the context of using genetic information to guide medical care. The use of genetic information in this manner arose from the Human Genome Project and technological advances that apply genomic information to medical practice (2).

Within genomic medicine, DNA sequence variations are mined for predictors of susceptibility and resistance to diseases, as well as for medication safety and efficacy. The former use has proven its utility in the diagnosis of many inherited disorders (3), the management of several cancers, and disease stratification (4). The latter has proven its usefulness for delineating appropriate anticancer therapies, anticoagulant therapy, and cholesterol reduction treatments among others (5, 6).

Genomic and precision medicine decisions generally rely on prior discovery and validation of genotype–phenotype associations across many patients. While this approach can be effective for patients with a previously identified disease correlation, it is inadequate for NIH UDP patients who present with undescribed problems. Herein, we describe the philosophical bases, methodologies, and processes that the NIH UDP developed to provide answers to patients with conditions that have eluded diagnosis and to advance biomedical knowledge about disease mechanisms.

## Defining Disease: The Philosophical Basis of the NIH UDP

As implied by the title UDP, definition of a diagnosis is crucial to understanding the Program’s purpose and approach. Given that a diagnosis is a culturally appropriate explanation for a problem (7), then, within Occidental medical culture, a diagnosis is a material and rational explanation testable by the scientific method (8). Within this perspective, diseases arise from malfunctioning biological processes causing harm and are not inclusive of illness caused by loss of mental or social well-being (9, 10). Adhering to this objectivist occidental medical definition, the NIH UDP has generally chosen to exclude diseases with sociocultural etiologies.

Biological or physiological malfunction is the product of gene–environment interactions over time (11). Thus, disease can be considered maladaptation to an ecological niche (12). Such maladaptations are characterized by disturbances of genetic, developmental, and physiological homeostases (12). The NIH UDP has defined genetic homeostasis as the sum of human evolutionary history encoded within DNA sequence, developmental homeostasis as the lifetime response of an organism to an ecological niche, and physiological homeostasis as the biochemical and molecular balance detectable at the moment of inquiry. In this construct, the developmental and physiological homeostatic responses to the environment are constrained by an organism’s genetic composition.

For most of human evolutionary history, natural selection molded humans to be hunter-gatherers. They walked many miles each day and ate a diverse, relatively unprocessed diet (11). Among many adaptations for survival, the development of culture sets humans apart from other organisms and allows them to change their environment to buffer against selective pressure. Through cultural evolution, humans colonize environments and develop lifestyles that they are not primarily adapted to by natural selection. Within the current urban lifestyle, for example, industrialization has exposed humans to novel toxins and processed food and enabled them to avoid most physical activity. Unable to alter millennia of natural selection, the mismatch of human bodies to this modern ecological niche causes most human disease in wealthy societies (11). These mismatch diseases, which include osteoporosis, cardiovascular disease, some cancers, type 2 diabetes, and metabolic syndrome, rarely arise from recent strong single-gene mutations but instead from multiple adaptations selected over the millennia of human existence. This perspective on gene–environment interactions consequently divides disease into rare monogenic or oligogenic disorders and common cultural mismatch disorders.

The NIH UDP has chosen for two reasons to focus its efforts on undescribed diseases likely to have a monogenic or oligogenic etiology. First, many cultural mismatch disorders are diagnosed and have defined etiologies and treatments (11). Second, monogenic or oligogenic disorders are more tractable for causal genetic discovery, and consequently, a material and rational explanation testable by the scientific method, i.e., a molecular diagnosis, is more achievable.

**Figure 1:**
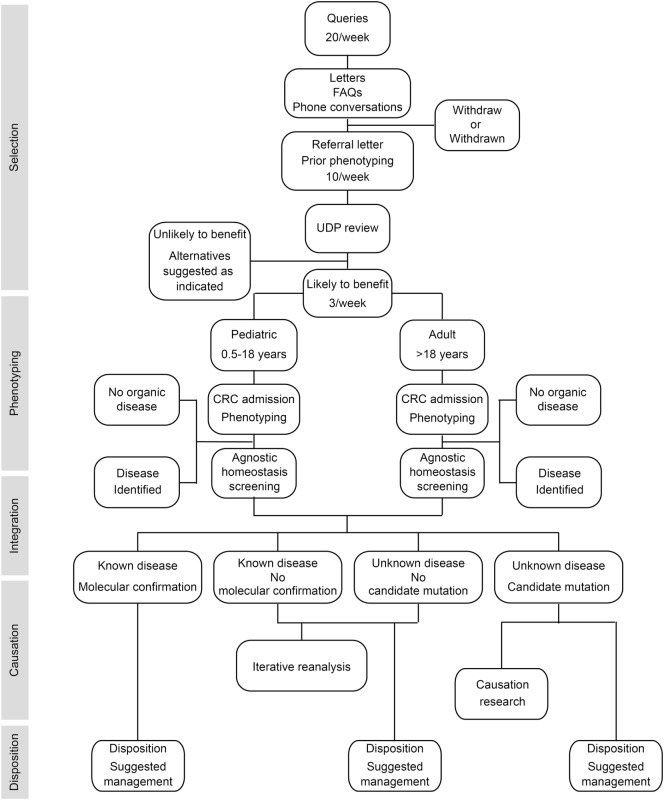
**Flow diagram showing the process by which patients are accepted into and evaluated for a diagnosis within the National Institutes of Health (NIH) Undiagnosed Diseases Program (UDP)**. The process is divided into five major components listed along the left side. The initial component is patient selection. This is followed by patient admission to the NIH clinical research center (CRC) for phenotyping and, when appropriate, agnostic screening for disturbances of evolutionary, developmental, and biochemical homeostases. These data are then integrated computationally and through discussion to determine if there is a known medical diagnosis. Patients with a diagnosis are given disposition recommendations based on that diagnosis. For those without a diagnosis and without a candidate cause, their data are queued for iterative reanalysis, and they and their referring physician are given disposition recommendations based on what was learned. For those without a diagnosis and with a candidate cause, their data are subjected, as resources allow, to research studies to evaluate the potential causality, and they and their referring physician are given disposition recommendations based on what was learned.

To provide answers for patients judged to have monogenic or oligogenic disorders that have eluded diagnosis, the NIH UDP screened for disturbances of the genetic, developmental, and physiologic homeostases. In addition, the NIH UDP implemented a management and communication system to facilitate collaborations and solutions (5). As represented in Figure 1, the NIH UDP process can be broken into the following steps: (1) patient selection, (2) patient phenotyping, (3) integrated analysis, (4) causal confirmation, and (5) disposition. The methodology and processes developed are described in the following sections.

## Methodologies and Results of the NIH UDP

### Patient Selection

Individuals with a broad spectrum of disorders apply to the NIH UDP (1, 13, 14). The experimental paradigm of the NIH UDP is predicated on an identifiable biological dysfunction arising from a monogenic or oligogenic etiology; thus, the ascertainment of the appropriate families is critical for an interpretable outcome and ethical experimentation (15–17). To satisfy these requirements, the NIH UDP selection criteria for admitting individuals to the clinical research center (CRC) included (1) a physician referral providing a clear picture of the patient’s illness and promising follow-up care after the UDP evaluation, (2) records of previous care and evaluations showing elimination of known disorders, (3) medical records and findings supporting a genetic etiology, (4) willingness of family members to participate for segregation of putative genetic causes, and (5) a problem within the expertise of the care available at the NIH CRC. All patients or their guardians and participating family members gave informed consent to clinical protocol 76-HG-0238, which was approved by the NHGRI Institutional Review Board.

### Patient Phenotyping

Having selected patients appropriate to the experimental paradigm, the next step for the NIH UDP was delineation of the disease phenotype. Given that disease is the loss of evolutionary, developmental, or physiological homeostasis and that a phenotype is the expression of that loss, characterization of the disease requires a thorough and unbiased assessment of each homeostatic disturbance. To this end, the NIH UDP implemented the following methodologies for assessment of these homeostases.

#### Assessment of Genetic Homeostasis

Nothing in biology makes sense except in the light of evolution.

Theodosius Dobzhansky

American Biology Teacher (1973) 35:125–129

Classically, genetic characterization has been performed by collecting a family history and carefully examining and testing family members to determine affected and unaffected status. This is usually presented as a pedigree and family history within the medical record. The NIH UDP continues this practice for immediate family members and occasionally for additional generations.

Given that the family and medical history meet criteria supporting a genetic etiology (see supplemental methods), identifying the point in the meiotic history of the family when the disturbance in evolutionary or genetic homeostasis occurred enables the generation of inheritance hypotheses and comparison of the affected individual’s genome to meiotically close reference genomes. Assessing evolutionary homeostasis through genome or exome sequencing, the NIH UDP developed and implemented DiploidAlign, an alignment strategy that imputes information from population and both parental genomes and then aligns the proband’s sequence data to those imputed genomes (see supplemental methods) (18–20).

#### Assessment of Developmental Homeostasis

Developmental homeostasis and its disturbances reflect the manifestation and evolution of disease during the lifetime of an individual. Classically, this information has been collected through medical history and serial physical examination. Although the temporal manifestation and evolution of disease are predicted to manifest in the transcriptome and epigenome profiles (21), the NIH UDP has not routinely assessed the transcriptome and epigenome because the disease-related changes were thought often specific to minimally accessible affected tissues.

Systematic collection of medical history and physical examination information require the use of a standardized vocabulary. Because traditional clinical vocabularies had been shown to be insufficient (22), the NIH UDP uses the Human Phenotype Ontology (HPO) (23), a standardized vocabulary of phenotypic abnormalities encountered in human disease, and the PhenoTips graphical user interface and search engine (24, 25). This allows comparison to other human disorders and model organisms as well as identification of relationships between human phenotypic abnormalities and cellular and biochemical networks (26).

#### Assessment of Physiologic Homeostasis

Physiologic or biochemical homeostasis reflects the equilibrium of the body at a moment in time, i.e., the moment at which the fluid or tissue is collected. Measurement of this homeostasis is the *sine qua non* of clinical pathology laboratories and is usually directed by a differential diagnosis. Given that the individuals presenting within the NIH UDP have, by definition, undescribed disorders, the differential diagnosis is absent to minimal, and thus, screens agnostic to diagnosis were used to detect disturbances of physiological or biochemical homeostasis.

Exemplifying the utility of this agnostic approach, approximately 50% of UDP patients screened for perturbation of protein glycosylation or free glycans in the plasma or urine differed from healthy controls (data not shown). These qualitative and quantitative changes in glycosylation, whether primary or secondary, have diagnostic, mechanistic, or therapeutic value as illustrated by detection of glycosylation abnormalities in DNA repair disorders (27, 28), ciliopathies (29, 30), mitochondriopathies (31, 32), and Golgi disorders (33). In contrast, detailed metabolomics studies uncovered very few anomalies, suggesting that the current medical testing technology already detects most disorders of metabolism prior to referral to the NIH UDP (data not shown). The NIH UDP did not pursue lipidome analysis; however, we hypothesize that, like the glycome analyses, these will define previously undetected primary and secondary changes having diagnostic, mechanistic, or therapeutic value and will be the subject of future investigation.

#### Integration of Measures of Homeostatic Disturbance

Having characterized these homeostases, the observations were integrated to minimize investigator bias and to generate testable hypotheses for disease causation. To accomplish this, the NIH UDP used the HPO terms to implement bioinformatic tools such as Exomiser^1^ and PhenIX (34–36). These software programs compare HPO terms to similar phenotypic profiles in humans and model organisms, improving prioritization of candidate disease variants. Illustrating the utility of this approach, reanalysis of UDP patient sequence data with Exomiser identified about 10–20% additional molecular diagnoses compared to those identified by manual curation alone (19).

This strategy also facilitated prioritizing of sequence variants within gene networks seeded by genes giving similar phenotypes when mutated in humans or model organisms and was effective for identifying atypical presentations (18, 37). A tool enabling such analysis is Exome Walker (38), which is incorporated into Exomiser for exome sequence analysis (19). This method prioritized mutations in *MED23* and *UNC80* as likely causes of neurodevelopmental disorders prior to mutations being reported in other families (19, 39, 40).

#### Delineation of a Sequence Variant As Causal or Not

In what circumstances can we pass from this observed association to a verdict of causation? Upon what basis should we proceed to do so?

Sir Austin Bradford Hill

Proceedings of the Royal Society of Medicine (1965) 58:295-300

Classically, a genetic cause for a trait is accepted if (1) variants segregate with disease, (2) multiple independent alleles of the gene give the same phenotype, and (3) expression of the wild-type gene rescues the phenotype. Accomplishment of these three in medical genetics is seldom possible. Consequently, medical genetics relies on associations that meet minimum evidence (41–43). In other words, causality in medical genetics is probabilistic and rarely deterministic (44).

Large pedigrees are generally used to acquire statistical evidence for segregation of a genetic locus with disease (43), and cohorts of independent families define independent alleles (45). When a disease occurs in a small family and is unrecognized or undescribed, characterization of segregation and identification of independent alleles is difficult. A proposed redress for both problems is to establish large databases of phenotypes and genotypes and use them to identify other families with the same disease and a shared potential genetic basis. To this end, the NIH UDP participates in the Matchmaker Exchange by depositing data in PhenomeCentral^2^ (46, 47). Data are also deposited in dbGaP.^3^ For some cases, we make a minimal amount of phenotypic and genotypic information available publically on the Monarch Initiative website^4^ to aid patient matching against known diseases and model organisms and to promote collaboration.^5^

In the absence of identifying another family, two methods can provide causative evidence: (1) amelioration of disease in the patient by pharmacologic targeting of the mutation or (2) recapitulation of the disease in a model system by introducing the precise mutation observed in the human. Exemplifying substantiation of causation through pharmacological targeting, a novel *de novo GRIN2A* mutation identified in a boy with early-onset epileptic encephalopathy was deemed as causative of his seizures, because the inhibitor identified *in vitro* for this mutant *N*-methyl-d-aspartate receptor effectively treated his seizures (48, 49). Illustrating delineation of causality through recapitulation of a disease in a model system, introduction of heterozygous *ATP6V1H* loss-of-function mutations in zebrafish and mice recapitulated the dominant osteoporosis segregating in the human family (50, 51).

In the absence of the above, the NIH UDP grades sequence variants to reflect the level of support. The first or lowest level is a bioinformatically derived likelihood of the variant being associated with disease. The second or intermediate level adds experimental evidence showing that the mutation alters properties of a gene product with a function consistent with the observed clinical phenotype. The third level adds *in vivo* studies to show overlap with the human phenotype and failure of the mutant but not of the wild-type human cDNA to rescue. Illustrating this third level of evidence, we used *Drosophila* model systems to carry out functional screens of 11 candidate genes to establish a causal link between rare genetic variants deemed potentially disease-causing and the nervous system phenotypes of UDP patients (Table 1). In the first phase of the screen, we used the *Drosophila* GAL4/UAS system to perform RNAi-mediated knockdown of candidate genes ubiquitously and specifically within the nervous system of *Drosophila* (52). We carefully analyzed effects on survival, behavior, and lifespan. Ubiquitous knockdown of each of the 11 genes resulted in early developmental lethality (Supplementary Table 1), suggesting that these genes are essential for viability. Also, nervous system-specific knockdown of each gene shortened adult life span and caused a degree of reduced developmental survival: mild (*DARS, SPRPK3, UBE2V2, MED23, GEMIN5*, and *NID2*), moderate (*CHD4* and *ATP1A3*), or severe (*AARS, GARS*, and *SMC3*). Analysis of the neural-motor circuit using negative geotaxis (climbing) behavior (53) detected moderate dysfunction with knockdown of DARS, MED23, and NID2 and more severe impairments with knockdown of UBE2V2 and SPRK3. Furthermore, for all knockdown groups, climbing behavior declined further at 20 days after eclosion, indicating a possible age-dependent impairment of CNS function.

**Table 1:** **Results of *Drosophila* central nervous system knockdown and rescue for 11 mutations in differing candidate genes**.

Patient	Human phenotype	Human gene	Fly homolog	*Drosophila* phenotype (CNS KD with C155)				Human expression construct	Rescue w/human cDNA	
	
				Survival index	Behavior (2 DAE)	Behavior (20 DAE)	Adult lifespan (LS_50_)		Rescue efficacy	Behavior Improvement (2 DAE)
5628	Spasticity, abnormality of the periventricular white matter, abnormality of the cerebral white matter, spinal cord lesions, impaired distal tactile sensation, impaired temperature sensation, impaired distal vibration sensation, Babinski sign, impaired distal proprioception, ankle clonus, knee clonus, gait disturbance, cataract	*DARS*	CG3821	0.89	♂ 37.4%, ♀ 50.4%	♂ 20.1%, ♀ 29.4%	♂ 68 DAE, ♀ 65 DAE	NM_001349.2:*wt*		
								c.839A>T; p.H280L	0.11	♂ 54.5, ♀ 41
								c.1099G>C; p.D367H	0.11	♂ 39.1, ♀ 32.1

4694	Specific learning disability, attention deficit hyperactivity disorder, ataxia, spasticity, morphological abnormality of the central nervous system, strabismus	*SRPK3*	CG11489	0.99	♂ 4.7%, ♀ 16.0%	♂ 0.06%, ♀ 0.02%	♂ 74 DAE, ♀ 72 DAE	NM_014370.3*:wt*	0.01	♂ 10.5, ♀ 44.1
								c.341G>A; p.G114E	0.01	♂ 89.1, ♀ 75.4
								c.475C>G; p.H159D	0/0.01	♂ 60.1, ♀ 58.1/♂ 10.1, ♀ 12.9
								c.1373C>A; p.T458N	−0.19/0.01	♂ 17.8, ♀ 16.7/♂ 5.3, ♀ −2.7

4245	Insomnia, chorea, elevated hepatic transaminases, malabsorption, gastric ulcer, iron deficiency anemia, elevated circulating catecholamine level, prolactin excess, palpitations, vomiting, bradycardia	*CHD4*	CG8103	0.5	NA	NA	NA	NM_001273.2:*wt*	0.50	Not compared
								c.4172G>A; p.G1391D	0.46	Not compared

2723	Ataxia, typical absence seizures, cerebellar atrophy, cerebral atrophy, dysarthria, short stature	*UBE2V2*	CG10640	0.98	♂ 0%, ♀ 0%	NA	♂ 5 DAE, ♀ 10 DAE	NM_003350:*wt*	−0.20	♂ 96.3, ♀ 83
								c.215A>C; p.K72T	−0.04	♂ 88, ♀ 75.8

2146, 2156	Profound global developmental delay, epileptiform EEG discharges, decreased muscle mass, hyperactive deep tendon reflexes, spastic tetraplegia, scissor gait, osteopenia, gait imbalance, elevated brain choline level by MRS, plagiocephaly, non-progressive encephalopathy, nasolacrimal duct obstruction, muscular hypotonia of the trunk, dystonia, chronic gastritis, choreoathetosis, Achilles tendon contracture, recurrent sinusitis, drooling, congenital cataract, chronic constipation, cerebral cortical atrophy, asthma, parietal bossing, flat occiput, EEG with focal slow activity, delayed myelination, coarse facial features, sleep disturbance, left-to-right shunt, irritability, Hashimoto thyroiditis, contractures of the joints of the lower limbs, pectus excavatum, recurrent otitis media, esotropia, ventricular septal defect, CNS hypomyelination, cerebral palsy, intellectual disability, severe	*MED23*	CG3695	0.99	♂ 46.0%, ♀ 55.7%	♂ 8.0%, ♀ 3.0%	♂ 42 DAE, ♀ 110 DAE	NM_015979.2:*wt*	0.01	♂ 3, ♀ 9
								c.3656A>G; p.H1219R	0.01	♂ −10.8, ♀ 27.4

3225	Abnormal protein *N*-linked glycosylation, low-set ears, anteverted nares, diffuse cerebral atrophy, cerebellar atrophy, dilation of lateral ventricles, abnormality of the cerebellar peduncle, scoliosis, rod-cone dystrophy, muscular hypotonia of the trunk, low CSF 5-methyltetrahydrofolate, EEG with generalized slow activity, cortical visual impairment, aplasia/hypoplasia of the cerebellum, EMG: neuropathic changes, neuronal loss in the cerebral cortex, hypoplasia of the pons, Dandy–Walker malformation, abnormality of the medulla oblongata, optic atrophy, abnormality of midbrain morphology, generalized hypotonia, cerebral white matter atrophy, generalized dystonia, exaggerated startle response, severe muscular hypotonia, poor head control, joint hypermobility, hyperactive deep tendon reflexes, bilateral single transverse palmar creases, bell-shaped thorax, ankle clonus, preauricular pit, myopathic facies, aspiration, short stature, decreased body weight, decreased fetal movement, cesarean section, postnatal microcephaly, growth delay, constipation, hip dysplasia, global developmental delay	*ATP1A3*	CG5670	0.27	Not analyzed (*n* = 4)	Not analyzed (*n* = 4)	Not analyzed (*n* = 4)	NM_152296.3:*wt*	0.03	*n* too small
								c.2408G>A; p.G803D	0.73	Not compared

4306	Birth length less than third percentile, memory impairment, depression, hand tremor, obsessive-compulsive behavior, bipolar affective disorder, aphasia, tachycardia, EMG: neuropathic changes, slow saccadic eye movements, periventricular leukomalacia, EEG with abnormally slow frequencies, exotropia, leukodystrophy, inappropriate behavior, urinary incontinence, recurrent urinary tract infections, dystonia, cogwheel rigidity, bowel incontinence, Babinski sign, abnormal conjugate eye movement, anxiety, aggressive behavior, expressive language delay, nystagmus, congenital strabismus, morphological abnormality of the central nervous system, spasticity, seizures, behavioral abnormality, intellectual disability, moderate	*AARS*	CG13391	0.09^a^	♂ 0%, ♀ 0%	NA	♀ 4 DAE (*n* = 10)	NM_001605.2:*wt*	0.84	♂ 94.3, ♀ 97.2
								c.242A>C; p.K81T	0.91	♂ 95, ♀ 91.6
								c.2251A>G; p.R751G	0.67	♂ 87.8, ♀ 89.8

5316	Decreased body weight, short stature, microcephaly, failure to thrive, intrauterine growth retardation, defect in the atrial septum, smooth philtrum, hypotelorism	*GARS*	CG6778	0^a^	NA	NA	NA	NM_002047.2:*wt*	0.68	Not compared
								c.246_249del; p.E83I(fs*6)	0.73	Not compared
								c.929G>A; p.R310Q	1.0	Not compared

3404	Sensorineural hearing impairment, pontocerebellar atrophy, laryngeal dystonia, postural instability, limb tremor, abnormal pyramidal signs, spastic dysarthria, progressive neurologic deterioration, myoclonus, oculomotor apraxia, abnormality of vision evoked potentials, vertical supranuclear gaze palsy, parkinsonism, mask-like facies, spasticity, ataxia, seizures, nystagmus, visual impairment	*GEMIN5*	CG30149	0.99	♂ 96.6%, ♀ 97.6%	♂ 96.1%, ♀ 88.6%	♂ 81 DAE, ♀ 96 DAE	NM_015465.4:*wt*	Did not perform rescue	
								c.2504A>G; p.K835R		

5509	Abnormality of the vertebrae, abnormality of the skeletal system, osteopenia, hypogammaglobulinemia	*SMC3*	CG9802	0.02^a^	NA	NA	<1 DAE	NM_005445.3:*wt*	0.98	Not compared
								c.3371C>A; p.A1124D	0.73	Not compared

1480, 1481	Global developmental delay, delayed fine motor development, delayed gross motor development, delayed speech and language development, intellectual disability, severe, autism, seizures, ataxia, dystonia, chorea	*NID2*	CG12908	0.99^a^	♂ 40.1%, ♀ 66.1%	♂ 42.9%, ♀ 24.8%	♂ 71 DAE, ♀ 76 DAE	NM_007361.3:*wt*	0.01	♂ 47, ♀ 9.6
								c.1904G>T; p.G635V	0.01	♂ 53.9, ♀ 26.1
								c.3887A>G; p.K1296R	−0.18	♂ 53.1, ♀ 12.3

*DAE, days after eclosion; NA, no adults; LS_50_, DAE of 50% of flies remaining; w/, with*.

*Survival index = (1 × percent survive to adult) + (0.5 × percent survive to pupae) + (0.25 × percent survive to larvae)*.

*Rescue efficacy: −1 = enhancement of KD survival phenotype, 0 = no rescue, 1 = suppression of KD survival phenotype*.

*^a^Survival index = 1 × percent survive to adult*.

*Behavior improvement: difference in percent climbing between rescue and RNAi KD at 2 DAE*.

In the second phase of this screen, we analyzed the consequence of overexpressing the human gene (wild-type or mutant variant) in flies with loss of the *Drosophila* ortholog. By using data from two independent experiments, we recorded enhancement or suppression of phenotypes associated with the loss of function in *Drosophila* (Table 1). Of the 11 genes, overexpression of six human wild-type genes (*ATP1A3, AARS, GARS, SMC3, NID2*, and *CHD4*) significantly suppressed loss-of-function phenotypes observed with CNS knockdown, suggesting the functional conservation between human and *Drosophila* orthologs. Comparing the rescue capability of the human wild-type versus the mutant constructs, expression of mutant constructs for three genes (*AARS, SMC3*, and *NID2*) had reduced rescue efficacy, expression of mutant constructs for two genes (*GARS* and *ATP1A3*) had greater rescue efficacy, and expression of mutant constructs for one gene (CHD4) showed no significant difference.

The reduced rescue efficacy of the human mutant versus wild type supported the pathological causality of the mutation, whereas neither a lack of difference between the mutant and wild type nor increased rescue efficacy of the mutant negated or supported causality. Possible explanations for a lack of difference between the mutant and wild type in the last two classes were that (1) the mutation is not deleterious; (2) the mutation is mildly deleterious and overexpression in *Drosophila* was sufficient to restore normal function; or (3) the mutation is deleterious, but the phenotype was below detection of the assay.

### Patient Disposition

Having completed these evaluations, a patient admitted to the NIH UDP might be diagnosed with a known disorder unrecognized during prior evaluations, an atypical presentation of a known disorder, a combination of several disorders, or a previously unreported disorder. Alternatively, in the absence of proof of causation or association, the patient’s problems might continue to elude explanation and remain undiagnosed. Disposition summaries, which are collaboratively decided on by the clinical and research staff, are communicated to all patients and their referring clinicians by letter and discussed by phone.

## Systems Management of the NIH UDP through Scalable Translational Research

The NIH UDP provides translational research for approximately 100–120 families per annum. Typically, individual physician scientists focus on a limited number of diseases; therefore, the NIH UDP defined a need for a scalable means of translational research.

Humans use the distribution of cognitive processes among a group with a variety of skills, e.g., a village, to solve complex problems. The success of these groups or villages requires knowledge of available resources, delineation of social relationships, and effective communication (54, 55). With this as precedent (56), the NIH UDP developed a scalable solution for translational research to coordinate the translational research needs of each family (5).

### Definition of a Common Knowledge Base

Performance of translational research requires knowledge and understanding of the problem: what has been tried to address the problem, what has been completed, and what reagents are available. To address these needs, the NIH UDP constructed an integrated system of inventory and data and process management, the Undiagnosed Diseases Program Integrated Collaboration System (UDPICS) (19). This system accumulates an inventory of all biospecimens and associated metadata at the time of collection. In addition, it collects and collates the information generated on each family during their clinical evaluation and research analysis. Finally, each temporal process is similarly documented and linked to antecedent and subsequent processes.

To facilitate coordination and communication, the NIH UDP implemented standard operating procedures, standardization of biospecimens and associated metadata, HPO description of patients (57), and standard genotypic and phenotypic description of model organisms.^6^ These provided a common foundation for solution generation and for data sharing.

### Building of Villages for Scalable Translational Research and Patient-Based Solutions

With delineation of available resources, the next challenge was addressing the patient or family’s problem. Historically, human hunter-gatherer and early agrarian communities formed around shared needs for survival. Modern communities continue to coalesce around shared values although not necessarily principles of survival. Unlike traditional physical villages, many modern communities are virtual and form through the use of Internet and social media tools. For both physical and virtual villages, member identification, communication, and a delineation of responsibilities and relationships are critical for community (58, 59).

In this context, the NIH UDP created virtual communities of geographically distributed experts to enable scalable translational research. Because identification of experts for such communities is traditionally limited by personal awareness, the NIH UDP also facilitated the development of computational tools using disease phenotypes or associated genotypes to identify and rank potential collaborators (60–62). These experts can be then contacted about collaborating on a patient’s problem. Although still a work in progress, UDPICS transformed translational research for the NIH UDP (19).

## Discussion

We report for the first time how the NIH UDP definition of disease within the rubric of evolutionary biology, i.e., maladaptation to an evolutionary niche, provided a logical construct for defining a systematic approach to diagnostic testing, interpretation, collaboration, and translational research. In this context, we also tested several theories including the alignment of next-generation sequence reads to deduced parental reference sequences, a systematic multistep approach to defining genetic causality for variants of uncertain significance, and distributed cognition as an efficient scalable model for translational research.

The embodiment of disease within the rubric of evolutionary biology and the delineation of the homeostatic components of adaptation allowed the NIH UDP, upon exhaustion of standard medical approaches, to take a systematic approach to agnostic measure of each of these homeostases. Chromosome microarray and exome sequencing, integrated with the phenome (*via* HPO) to measure both evolutionary and developmental homeostasis, detected diagnostic mutations in 20–30% of patients (1, 63). These measures, integrated with agnostic analyses of the glycome, seem poised to delineate disease mechanisms and causes in possibly half of the NIH UDP patients.

Postulating that mutations causing undiagnosed disorders are missed because haploblock-specific variants impede sequence alignment, we tested measurement of evolutionary homeostasis by aligning patient sequence to parental- and population-deduced reference sequences. This approach marginally improved alignment and genotyping to Hg37 but did not increased diagnostic rate. Therefore, although this approach is cognizant of the diploid nature of the human genome and haploblock-specific variants, it does not appear to improve detection of causal mutations sufficiently to justify the increased computational costs. In contrast, based on the preliminary studies of others, *de novo* assembly of long reads might enable detection of causal mutations undetected by short read next-generation sequencing (64).

Delineation of causative variants for traits investigated within individualized precision medicine remains problematic (65–69). The NIH UDP experience and the conclusions of others reiterate prior tenets of medical genetics regarding definition of causality. Specifically, the NIH UDP has observed that, for a single individual, defining a variant in a gene not previously associated with a trait as causative of that trait is not scientifically defensible. As stated by MacArthur et al. “strong evidence that a variant is deleterious (in an evolutionary sense) and/or damaging (to gene function) is not sufficient to implicate a variant as playing a causal role in disease” (65). In contrast, delineation of causality for a novel variant in a gene previously associated with a trait is possible as we demonstrated herein using pharmacologic suppression and *Drosophila melanogaster* as a model system.

Identifying pathogenicity for the many different novel variants identified in disease-associated genes requires collaboration to leverage global expertise. The underlying principles for this are those of distributed cognition (54, 55) enabled through various Internet and social media tools (19). By this means, the NIH UDP was able to systematically and methodically assemble virtual villages of collaborators to provide translational research appropriate to each problem and to provide medically and economically efficient translational research.

We conclude that the NIH UDP experience of systematically and methodically integrating concepts from multiple disciplines provides a guide for individualized or personalized medical practices. These principles are currently being refined and extended through the Undiagnosed Diseases Network launched by the NIH in September 2015 (63) and through the Undiagnosed Diseases Network International (70).

## Ethics Statement

All patients or their guardians and participating family members gave informed consent to clinical protocol 76-HG-0238, which was approved by the NHGRI Institutional Review Board.

## Author Contributions

WG, DA, TM, RZ, and CFB conceived the methodology. WG, DA, TM, JD, RG, CG, DD, GG, MN, CW, LW, CJT, and CT participated in patient selection and phenotyping. MAH, PR, D Smedley, and NW developed the HPO annotation guidelines, curated the patients’ phenotype data, and helped with the exome analysis. EV, TG, CA, WB, AB, LC, EF, JG, JE, PP, D Simeonov, EL, AL, D Smedley, MS, KX, DA, and TM participated in patient genotyping, the development of DiploidAlign, or implementation of DiploidAlign. CB, JB, MD, ZD-P, YH, MK, CL, VM, MM, FM, JP, KS, YZ, and RZ performed cell biology, biochemical, and model organism experiments to delineate causality. RF, YH, and VM participated in patient sample extraction, archiving, and distribution. EV, CA, WB, DD, EF, JG, EL, AL, VM, KS, MS, and ZV developed or implemented and tested software infrastructure. EV, CFB, CB, and RZ wrote the manuscript. All authors read and approved the final manuscript.

## Conflict of Interest Statement

The authors declare that the research was conducted in the absence of any commercial or financial relationships that could be construed as a potential conflict of interest.

## Footnotes

^1^https://exomiser.github.io/Exomiser/.

^2^https://phenomecentral.org/.

^3^http://www.ncbi.nlm.nih.gov/gap.

^4^www.monarchinitiative.org.

^5^https://beta.monarchinitiative.org/case/MONARCH:c000001#overview.

^6^http://www.phenote.org/.
