# Supplementary material for: Defining Disease, Diagnosis, and Translational Medicine within a Homeostatic Perturbation Paradigm: The National Institutes of Health Undiagnosed Diseases Program Experience
Source: Front Med (Lausanne). 2017 May 26;4:62. doi: 10.3389/fmed.2017.00062 (PMC5445140; doi:10.3389/fmed.2017.00062)

Defining disease, diagnosis, and translational medicine within a homeostatic perturbation paradigm: The NIH Undiagnosed Diseases Program experience

Timothy Gall[1,3]*, Elise Valkanas[1]*, Christofer Bello[2]*, Thomas Markello[1], Christopher Adams[1], William Bone[1], Alexander J. Brandt[1], Jennifer M. Brazill[2], Lynn Carmichael[4], Mariska Davids[1], Joie Davis[1], Zoraida Diaz-Perez[2], David Draper[1,3], Jeremy Elson[5], Elise Flynn[1], Rena Godfrey[1], Catherine Groden[1], Cheng-Kang Hsieh [5], Roxanne Fischer[3], Gretchen A. Golas[1], Jessica Guzman[1], Yan Huang[1], Megan S. Kane[1], Elizabeth Lee[1], Chong Li[2], Amanda E. Links[1], Valerie Maduro[1], May Christine V. Malicdan[1], Fayeza S. Malik[2], Michele Nehrebecky[1], Joun Park[2], Paul Pemberton[1], Katherine Schaffer[1], Dimitre Simeonov[1], Murat Sincan[1], Damian Smedley[6], Zaheer Valivullah[1], Colleen Wahl[1], Nicole Washington[7], Lynne A. Wolfe[1,3], Karen Xu[1], Yi Zhu[2], William A. Gahl[1,3], Cynthia J. Tifft[1,3], Camillo Toro[1], David R. Adams[1,3], Miao He[8], Peter N. Robinson[9], Melissa A. Haendel[10], R. Grace Zhai[2], Cornelius F. Boerkoel[1]

[1]NIH Undiagnosed Diseases Program, Common Fund, Office of the Director, National Institutes of Health, Bethesda, MD, USA

[2]Department of Molecular and Cellular Pharmacology, University of Miami School of Medicine, Miami, FL, USA

[3]National Human Genome Research Institute, National Institutes of Health, Bethesda, MD, USA

[4]Appistry, Inc., St. Louis, MO, USA

[5]MicroSoft Research, Redmond, WA, USA

[6]Palmieri Metabolic Disease Laboratory, Children's Hospital of Philadelphia, Philadelphia, PA; Department of Pathology and Laboratory of Medicine, University of Pennsylvania, Philadelphia, PA, USA

*These authors contributed equally to this work

Correspondence:

David Adams, MD, PhD,
10 Center Drive, MSC 1851,
10/10C-103,
NHGRI, NIH,
Bethesda, Maryland 20892-1851,
Phone 301-402-6435,
FAX 301-402-7290,
Email: dadams1@mail.nih.gov

Supplementary Materials, Methods and Data

NIH UDP approach for screening patients for a likely genetic etiology to disease

Data that suggest an acquired disorder:

a. Normal state of health, with no evidence of atypical physiology up to a very defined event that can be linked to the subsequent medical problem - specially when there is a specific major infection or vascular accident or episode of hypoxia prior to the start of the phenotype that is undiagnosed.
b. No pedigree evidence of inheritance in this case or others with similar phenotypes in any literature.
c. Single anatomical or developmental system is the only involved system, especially one that is very general (cognitive dysfunction, weakness, single limb abnormality not seen in other limb)
d. Anything that involves the skin, the immune system, arthritis, seizures or pain especially if a single triggering event evokes the symptomatology, and that trigger would likely to have been present earlier in life when the problem was not present.
e. In vitro pregnancies, multiple gestation with one affected only.
f. General medical consensus by referring physicians that this is most probably an acquired condition.

Data that suggests a genetic disorder:

a. A family pedigree that describes at least one other member of the pedigree that has credible evidence of the same medical condition.
b. Multiple affected systems in the body.

c. Symptoms recognized or present since birth and stay atypical throughout life
d. Phenotypes that are part of described developmental systems (size, bone morphology)
e. Metabolic evidence for a dysfunction (pigmentary changes, failure to thrive)
f. Plausible connection to a cell signaling pathway
g. Structural evidence of a metabolic active cell type: e.g., retina, kidney tubule, extra ocular muscles, Purkinje cells, cardiac myopathy (especially hypertrophy).
h. Cellular inclusions on biopsy
i. Abnormal developmental fields: Pharyngeal clefts/ridges, midface dysmorphology, nasopharyngeal clefts, tracheolaryngeal malformations, cervical vertebral anomalies, costochondral/clavicular maldevelopment, laterality defects of the thorax or abdomen, embryological defects of growth (abdominal wall defects, hypoplastic limbs, genital hypoplasia), ray defects – (extra/missing toes)

<u>NIH UDP additional considerations for medical utility of exome or genome sequencing</u>

Features of disease or problem (severity or uniqueness) suggesting utility:

a. Major functional loss
b. Unique findings on biopsy that are new and unique when reviewed by experts
c. Progressive disease
d. Threat to life if progressive
e. Intervention possible
f. Other family members/ others in population at risk too

g. Major disease in the population that would benefit from multifactorial dissection by identifying at least one (more) genetic cause

Features of disease or problem (severity or uniqueness) suggesting a lack of utility:

a. Non-vital single system (e.g., toe nail discoloration of only one foot)
b. No vital loss of function (i.e., easily adaptable medical condition)
c. Patient has dramatically improved or adapted to condition when assessed at the NIH visit (e.g., speech delay that corrected, gait disturbance that has lessened)

Features of disease or problem (severity or uniqueness) suggesting a potential viable assay for determination of causation:

a. Established cell line from the patient that shows a chemical or morphological change from cells, especially skin, lymphoblast or some easily transformed IPS line
b. Biochemical serum/plasma/urine/CSF value
c. Mitochondrial inefficiency
d. Published sensitivity of a candidate gene to a toxin/ infectious agent
e. A model organism exists for the problem

Features of disease or problem (severity or uniqueness) suggesting a lack of a viable assay for determination of causation:

a. Anything that would be a subjective symptom with n=1
b. Most symptoms that are highly sporadic and unpredictable when they occur (i.e., a weakness that has occurred for 24 hour once every 3 to 5 years not periodically)

c. Symptoms that have only been seen by the patient or a parent and has only been witnessed in a single location

d. Any symptom that would take observations (e.g., MRI scans) over many years in duration or monitoring (e.g., EEG or EKG) for prolonged periods

e. Most things that would require very invasive biopsies (brain, spinal cord, lung, heart, liver)

<u>NIH UDP scoring rubric for eligibility for exome or genome sequencing</u>

Pre-Screening I: General Variable Attributes

1. Is the case likely to be genetic (early age of onset, developmental pattern, multiple affected individuals, etc.)?

1	2	3	4	5
Acquired				Genetic

2. Is the case interesting enough to be worth extensive genetic testing and analysis (severe, rare/unique, treatable, etc.)?

1	2	3	4	5
Mild, overlaps with common conditions, no imaginable treatment				Unique/Rare, compelling, treatments imaginable

3. Is there some feasible way to test/know whether any given candidate variant is the disease-causing variant?

1	2	3	4	5
Neuro phenotype with normal structural/functional studies, no unifying mechanism, diverse disease processes				Cell phenotype, metabolic marker, developmental pattern, energy metabolism marker

Pre-Screening II: General Exclusion/Inclusion Questions

1. Are there clinical or other clues that could focus attention on a specific chromosomal region or set of genes? If autosomal dominant (not *de novo* dominant) or if family structure score is low, consider sending single exome for assay of known candidate genes.
2. Is it possible to perform adequate phenotyping for all family members? Family members that cannot be phenotyped (including conditions with suspected incomplete penetrance or family members who are younger than the typical age of onset) should be excluded from the analysis.
3. Is there reason to suspect consanguinity? If so, consider running SNP chip on affected person to look for evidence of consanguinity (regions of homozygosity). In some cases, a single SNP chip and a single exome or genome can be used to solve such cases. This also applies if pre-exome SNP analysis shows the same.
4. Does the case suggest that non-coding DNA, RNA, epigenetic phenomena or other mechanism is likely enough that additional types of screening analysis should be considered (methylome, transcriptome, ChIP-chip, targeted capture, etc.)? If transcriptome analysis is considered, is an appropriate tissue available and/or is the case interesting enough to warrant making pluripotent stem cells? If so, consider bringing up at UDP lab meeting to design custom plan for investigation.

Pre-Screening III: Adequacy of Available Specimens Given Genetic Model

(only count individuals with unambiguous phenotypes)

1. Autosomal Recessive Inheritance Possible

1	2	3	4	5
Two unaffected parents, one affected child	Two unaffected parents, one affected child, 1+ unaffected child	Two unaffected parents, 1 affected child, 1+ unaffected child, additional affected family members	2 affected children of same parents	>2 affected children of same parents or 2 affected and 1+ unaffected child of same parents
1	2	3	4	5
Single individual's DNA available	Affected plus one related individual's DNA available	DNA from affected, one parent, two unaffected sibs, or both-parent trio	DNA from both parents, two affected children	DNA from both parents, 2+ affected, and 1+ unaffected

2. New Dominant Inheritance

1	2	3	4	5
Single individual	One parent, one unaffected sib, one affected sib	Trio, both parents and affected, or one parent plus affected plus 2+ unaffected sibs	As with situation 3, but additional family members have disease in dominant-appearing pedigree	Unaffected (I) generation, affected (II) generation, affected (III) from affected (II)
1	2	3	4	5
Single individual	DNA from situation 2 above, all available	DNA from situation 3 above, all available	DNA from situation 3 above, all available	DNA from situation 5 above available for unaffected grandparent and one affected

3. X-Linked (~recessive) Inheritance

1	2	3	4	5
Unaffected mother, single affected boy	Unaffected mother, two affected boys	Unaffected mother, two affected boys	Unaffected mother, 2+ affected boys, plus supportive pedigree or (+) X inactivation study	Unaffected mother, 2+ affected boys, plus supportive pedigree or (+) X inactivation study
1	2	3	4	5
DNA from mother, affected boy	DNA from mother, 2+ affected boys	DNA from mother, affected boys, 1+ unaffected boys	DNA from mother, 2+ affected boys	DNA from mother, affected boys, 1+ unaffected boys

4. Dominant Inheritance

1	2	3	4	5
Single affected individual	Parent child pair of affected individuals	Large pedigree c/w AD inheritance, <3 LOD Score		Large pedigree, positive linkage study
1	2	3	4	5
DNA from single affected individual only	DNA from affected parent and affected offspring	DNA from affected parent and affected offspring and 1+ unaffected offspring	DNA from 2+ affected parent-child trios from distant family branches	DNA from all critical meioses in positive linkage family

Scoring system: use best score from among valid genetic models

Pre-Screening I: >= 9 equals pass, otherwise defer case

Pre-Screening II: Record answers, consider individually

Pre-Screening III: >= 6 equals pass, otherwise defer case

<u>NIH UDP exome short read alignment, genotyping and analysis</u>

The DiploidAlign custom alignment strategy was developed to increase alignment accuracy and improve genotype calling by creating a modified reference sequence that more closely resembles a patient's true genomic sequence. The DiploidAlign method consists of four parts: reference creation, alignment, liftover, and genotype calling.

Reference Creation

Two customized haploid reference sequences were created for each individual by imputing genetic data orthogonal to the NGS read data. Genomic SNP microarray data from the patient and both parents were curated for high quality calls (infinium2vcf). Briefly, we mapped the Illumina array probe sequences to the hg19 genome assembly using the Burrows-Wheeler Aligner (BWA) and excluded SNPs with non-specific probe alignment, those with common variants near the 3-prime end of probe alignments, and those where neither of the assayed alleles matched the reference. We converted the Illumina data using these mapped coordinates; the resulting SNPs were then phased using family pedigree information and additional SNP and INDEL information was imputed using HapMap data from the 1000 Genomes Project (Beagle v4)[1-3]. From these data, we created two modified versions of the human reference sequence hs37d5 (ftp://ftp.1000genomes.ebi.ac.uk/vol1/ftp/technical/reference/phase2_reference_assembly_sequence) for each individual, each version representing a haploid version of the individual's diploid genome (vcf2diploid AlleleSeq)[4]. These data and vcf2diploid (AlleleSeq) were used to create two modified versions of hs37d5, one including maternally inherited variation and the other paternally inherited variants. These two haploid reference sequences were then concatenated to create a third, parental reference sequence to be used for resolving alignment differences between the two sequences.

Sequencing and Alignment

Whole blood DNA was prepared with the Illumina TruSeq Exome Enrichment Kit and sequenced on Illumina HiSeq 2000 to generate 101 bp paired end read sequences.

All reads from each patient were aligned to the modified reference sequences separately: once to the maternal reference, once to the paternal reference, and once to the concatenated parental reference using Novalign (http://www.novocraft.com/).

Liftover

Based on the resulting positions and MAPQ scores in the three alignments, each read was assigned to either the maternal or paternal haplotype. A custom extension of Picard Liftover Java class (http://picard.sourceforge.net) was used to resolve positions and MAPQ scores between the different alignments, translate coordinates to hs37d5, and reformat irregular CIGAR strings.

Briefly, the chromosome to which the read aligned in the parental alignment determined whether the read was assigned to the maternal or paternal haplotype. The read was then assigned the position and MAPQ score from that alignment (maternal or paternal). However, if a read aligned with a MAPQ score of 0 in the parental alignment (i.e. the read matched equally well to two positions on the concatenated parental reference sequence), the read was assigned the position and MAPQ score from the maternal or paternal alignment with the lower MAPQ score. Reads that had the same MAPQ score in both alignments were randomly assigned to a haplotype.

Following resolution of reference sequence, the read positions were translated from the modified reference sequence to the standard hs37d5 reference sequence using a standard liftover procedure. Irregular CIGAR strings following liftover were reformatted using Smith-Waterman alignment[5].

Genotype Calling

The single reformatted alignment was processed using Best Practice guidelines[6,7] from the Genome Analysis Toolkit version 3.1.7[8], including MarkDuplicates (Picard), Indel Realignment (GATK), and Base Recalibration (GATK). The processed alignment was then genotyped and variant called using GenotypeGVCFs and HaplotypeCaller to generate a VCF file with non-reference variation.

Evaluation

To evaluate performance, DiploidAlign was compared to a standard alignment strategy, i.e., one that aligned reads to the standard hs37d5 reference sequence without modification, and differences were interrogated by Sanger sequencing. Both strategies were performed on the same 70 individuals (18 families) from the UDP cohort and on the NA12878 trio (ftp://ftp-trace.ncbi.nlm.nih.gov/giab/ftp/data/NA12878/NIST_NA12878_HG001_HiSeq_300x) reprocessed in house. DiploidAlign takes approximately four times more computational time than the standard alignment process, but results in an increased number of mapped reads, an increase in gapped alignments following liftover (as a proxy for correctly placed INDELS), and improved genotype accuracy as evaluated by Sanger sequencing.

<u>NIH UDP analysis of novel mutations using transgenic *Drosophila*</u>

Fly stocks and culture: Flies were maintained on a cornmeal–molasses–yeast medium and at room temperature (22°C) with 60–65% humidity. The RNAi lines were obtained from either Bloomington Stock Center or Vienna Drosophila Resource Center.

For making transgenic flies, human cDNA plasmids were obtained from UDP and sent to Genetic Services and Gentivision to generate transgenic stocks.

Negative geotaxis behavior assay

Negative geotaxis behavior (climbing) assay was modified from the method originally described by Benzer [9]. The detailed method is described in Ali et al [10]. Ten age/gender-matched flies of the same genotype were placed in a vial marked with a line drawn horizontally 8 cm above the surface. The flies were gently tapped to the bottom surface and given 10 s to demonstrate climbing activity as a negative geotaxic response. After 10 s, the number of flies that successfully climbed above the 8-cm line was recorded. This assay was repeated 10 times and the averaged data were represented as percentages where the number of flies above the 8-cm mark was divided by the total number of flies tested within each group.

References

1 Browning, S. R. & Browning, B. L. Rapid and accurate haplotype phasing and missing-data inference for whole-genome association studies by use of localized haplotype clustering. *American journal of human genetics* **81**, 1084-1097, doi:10.1086/521987 (2007).

2 Browning, B. L. & Browning, S. R. Genotype Imputation with Millions of Reference Samples. *American journal of human genetics* **98**, 116-126, doi:10.1016/j.ajhg.2015.11.020 (2016).

3 Browning, B. L. & Browning, S. R. Improving the accuracy and efficiency of identity-by-descent detection in population data. *Genetics* **194**, 459-471, doi:10.1534/genetics.113.150029 (2013).

4 Rozowsky, J. *et al.* AlleleSeq: analysis of allele-specific expression and binding in a network framework. *Molecular systems biology* **7**, 522, doi:10.1038/msb.2011.54 (2011).

5 Smith, T. F. & Waterman, M. S. Identification of common molecular subsequences. *Journal of molecular biology* **147**, 195-197 (1981).

6 DePristo, M. A. *et al.* A framework for variation discovery and genotyping using next-generation DNA sequencing data. *Nature genetics* **43**, 491-498, doi:10.1038/ng.806 (2011).

7 Van der Auwera, G. A. *et al.* From FastQ data to high confidence variant calls: the Genome Analysis Toolkit best practices pipeline. *Current protocols in bioinformatics* **43**, 11 10 11-33, doi:10.1002/0471250953.bi1110s43 (2013).

8 McKenna, A. *et al.* The Genome Analysis Toolkit: a MapReduce framework for analyzing next-generation DNA sequencing data. *Genome research* **20**, 1297-1303, doi:10.1101/gr.107524.110 (2010).

9 Benzer, S. Genetic dissection of behavior. *Scientific American* **229**, 24-37 (1973).

10 Ali, Y. O., Escala, W., Ruan, K. & Zhai, R. G. Assaying locomotor, learning, and memory deficits in Drosophila models of neurodegeneration. *Journal of visualized experiments : JoVE*, doi:10.3791/2504 (2011).

Supplementary Table 1. Results of *Drosophila* ubiquitous knockdown and rescue for 11 mutations in differing candidate genes.

Gene	Fly Homologue	Drosophila Phenotype (Ubiquitous KD with Actin)		Human Mutation	Rescue w/ Human cDNA	
		Survival Index	Adult Lifespan (LS_{50})		Rescue Efficacy	Adult Lifespan (LS_{50})
DARS	CG3821	0.25	NA	NM_001349.2:*wt*		
				c.839A>T; p.H280L	0.75	♂ 77 DAE, ♀ 77 DAE
				c.1099G>C; p.D367H	0.75	♂ 78 DAE, ♀ 76 DAE
SRPK3	CG11489	0.46	NA	NM_014370.3:*wt t*	0.05	not analyzed (n=2)
				c.341G>A; p.G114E	0.04	NA
				c.475C>G; p.H159D	0.24 / 0.05	♂ 135 DAE, ♀ 103 DAE / not analyzed (n=6)
				c.1373C>A; p.T458N	0.11 / 0.06	♂ 123 DAE (n=9), ♀ 85 DAE (n=8) / not analyzed (n=14)
CHD4	CG8103	0.25	NA	NM_001273.2:*wt*	0.28 / 0.01	not analyzed (n=6) / NA
				c.4172G>A; p.G1391D	0.25	not analyzed (n=2)
UBE2V2	CG10640	0.47	NA	NM_003350:*wt t*	0.03	NA
				c.215A>C; p.K72T	0.03	NA
MED23	CG3695	0.3	NA	NM_015979.2:*wt*	0.08	not analyzed (n=6)
				c.3656A>G; p.H1219R	0.01	not analyzed (n=4)
ATP1A3	CG5670	0.26	NA	NM_152296.3:*wt*	0	NA
				c.2408G>A; p.G803D	-0.26	NA
AARS	CG13391	0.03#	NA	NM_001605.2:*wt*	-0.03#	NA
				c.242A>C; p.K81T	-0.03#	NA
				c.2251A>G; p.R751G	-0.03#	NA
GARS	CG6778	0#	NA	NM_002047.2:*wt*	0#	NA
				c.246_249del; p.E83I(fs*6)	0#	NA
				c.929G>A; p.R310Q	0#	NA
GEMIN5	CG30149	0.06#	NA	NM_015465.4:*wt t*	-0.06#	NA
				c.2504A>G; p.K835R	-0.06#	NA
SMC3	CG9802	0#	NA	NM_005445.3:*wt*	0#	NA
				c.3371C>A; p.A1124D	0#	NA
NID2	CG12908	0.26#	NA	NM_007361.3:*wt*	0.28#	♀ 49 DAE
				c.1904G>T; p.G635V	-0.26 / 0.33#	NA / ♀ 79 DAE
				c.3887A>G; p.K1296R	0.27#	♀ 65 DAE

DAE = Days After Eclosion
NA = no adults
Survival Index = (1 X percent survive to adult) + (0.5 X percent survive to pupae) + (0.25 X percent survive to larvae)
#Survival Index = 1 X percent survive to adult
LS_{50} = DAE of 50% of flies remaining
Rescue Efficacy : -1 = enhancement of KD survival phenotype, 0 = no rescue, 1 = suppression of KD survival phenotype
Behavior Improvement : difference in percent climbing between rescue and RNAi KD at 2 DAE

Figure legends

Supplementary Figure 1.

Flow diagram comparing the DiploidAlign pipeline and standard pipeline used to align and gentoype whole exome sequencing data at the NIH UDP. The DiploidAlign process depicts use of family SNP array and population HapMap data to create three separate reference sequences: two haploid reference sequences (maternal and paternal) and one concatenated diploid reference sequence (parental). Short reads from exome sequencing are independently aligned to each reference sequence, and a custom liftover process resolves all three alignments into a single alignment with coordinates corresponding to the standard reference sequence. The standard alignment pipeline uses the same aligner, but does not incorporate SNP array or population data to align short reads from exome sequencing. Both pipelines perform the same post-alignment processing, which consists of base and variant recalibration as well as genotyping.

Supplementary Figure 2.

Results of DiploidAlign pipeline versus standard alignment pipeline. A. Short read mapping statistics. There was a statistically significant increase in mapped reads, high quality mapped reads, and gapped alignments post-liftover, and a statistically significant decrease in gapped alignments pre-liftover between pipelines. B. Results of Sanger sequencing validation of variant calls. Of 147 high-quality genotype differences, Sanger sequencing verified DiploidAlign genotype calls more often than standard alignment (one sample test of proportions, p = 1x10 -8). The Sanger sequencing results verified DiploidAlign 79% of the time for SNPs and 58% of the time for INDELs.

Supplementary Figure 1

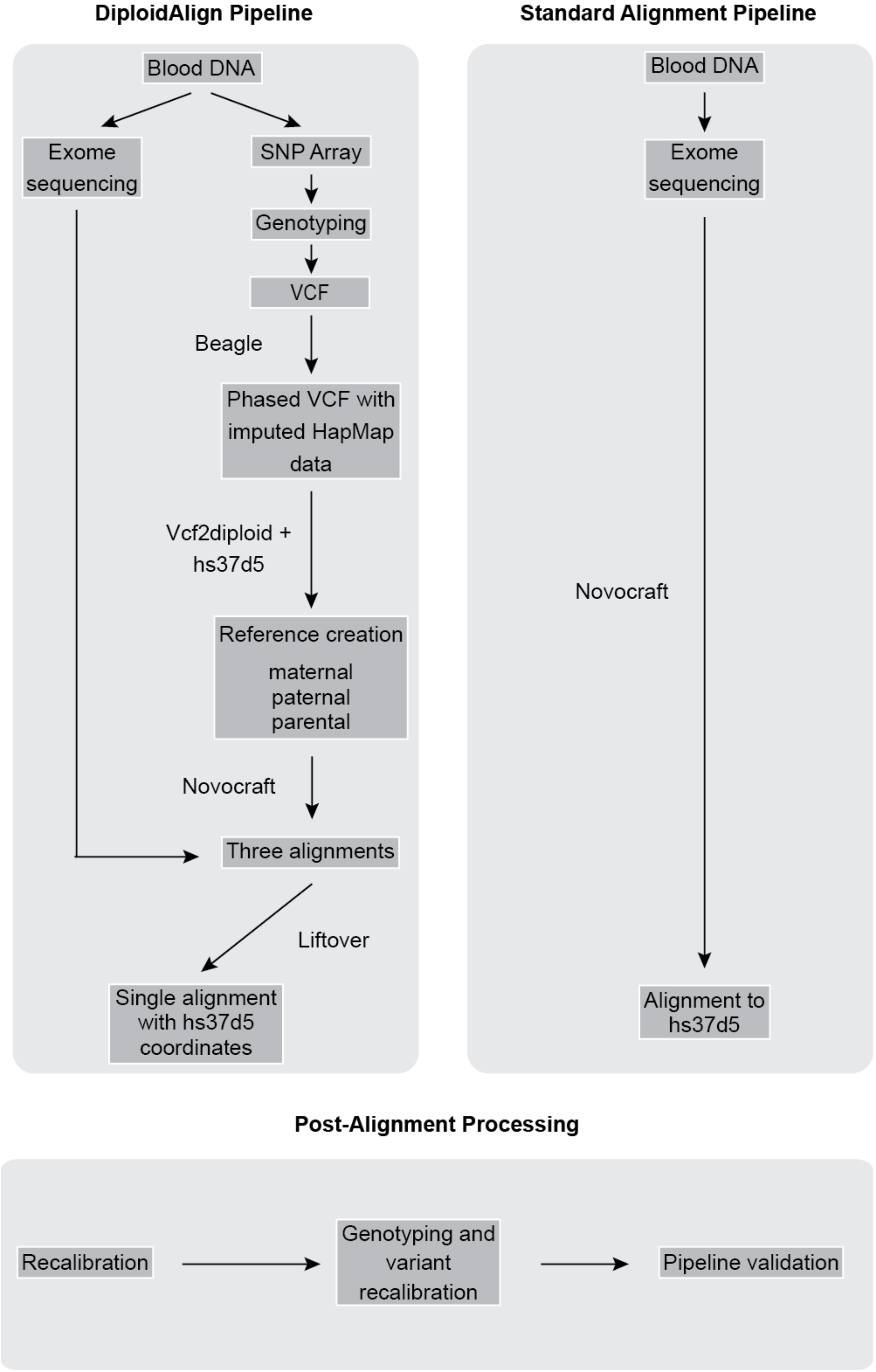

Supplementary Figure 2

A

	Standard	DiploidAlign	Percent Change from Standard	p-value	Min	Max
Mapped Reads	$1.7x10^8$ ± 0.1	$1.7x10^8$ ± 0.1	0.007%	7E-18	0.002%	0.014%
High Quality Mapped Reads (MAPQ >= 30)	$1.5x10^8$ ± 0.1	$1.5x10^8$ ± 0.1	0.014%	2E-09	-0.012%	0.043%
Gapped Alignments: Pre-Liftover	$1.4x10^6$ ± 0.1	$1.1x10^6$ ± 0.1	-26.511%	5E-24	-29.3%	-13.7%
Gapped Alignments: Post-Liftover	$1.4x10^6$ ± 0.1	$1.5x10^6$ ± 0.1	5.83%	2E-23	2.7%	7.0%

B

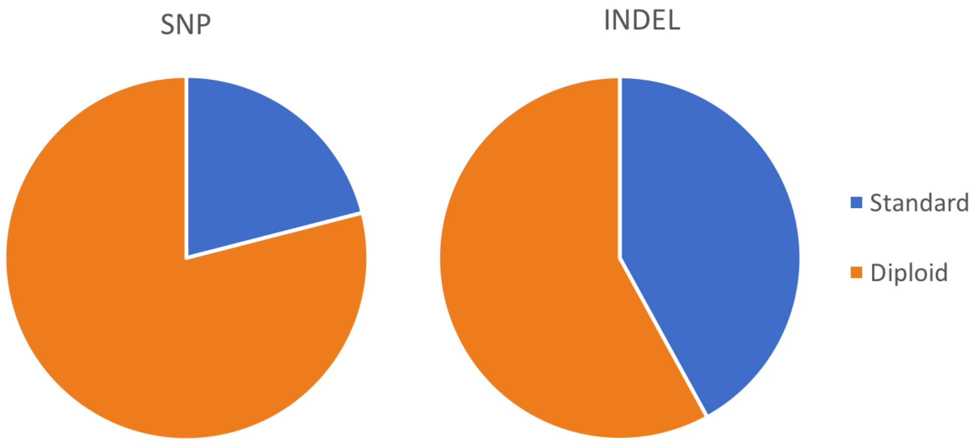

Supplement: Supplementary file 1 [file Data_Sheet_1.PDF]
